# Evidence that the intra-amoebal *Legionella drancourtii *acquired a sterol reductase gene from eukaryotes

**DOI:** 10.1186/1756-0500-2-51

**Published:** 2009-03-27

**Authors:** Claire Moliner, Didier Raoult, Pierre-Edouard Fournier

**Affiliations:** 1 URMITE CNRS-IRD UMR 6236, Faculté de Médecine, 27 boulevard Jean Moulin, 13385 Marseille, Cedex 05, France

## Abstract

**Background:**

Free-living amoebae serve as a natural reservoir for some bacteria that have evolved into «amoeba-resistant» bacteria. Among these, some are strictly intra-amoebal, such as *Candidatus *"Protochlamydia amoebophila" (*Candidatus *"P. amoebophila"), whose genomic sequence is available. We sequenced the genome of *Legionella drancourtii *(*L. drancourtii*), another recently described intra-amoebal bacterium. By comparing these two genomes with those of their closely related species, we were able to study the genetic characteristics specific to their amoebal lifestyle.

**Findings:**

We identified a sterol delta-7 reductase-encoding gene common to these two bacteria and absent in their relatives. This gene encodes an enzyme which catalyses the last step of cholesterol biosynthesis in eukaryotes, and is probably functional within *L. drancourtii *since it is transcribed. The phylogenetic analysis of this protein suggests that it was acquired horizontally by a few bacteria from viridiplantae. This gene was also found in the *Acanthamoeba polyphaga Mimivirus *genome, a virus that grows in amoebae and possesses the largest viral genome known to date.

**Conclusion:**

*L. drancourtii *acquired a sterol delta-7 reductase-encoding gene of viridiplantae origin. The most parsimonious hypothesis is that this gene was initially acquired by a *Chlamydiales *ancestor parasite of plants. Subsequently, its descendents transmitted this gene in amoebae to other intra-amoebal microorganisms, including *L. drancourtii *and *Coxiella burnetii*. The role of the sterol delta-7 reductase in prokaryotes is as yet unknown but we speculate that it is involved in host cholesterol parasitism.

## Background

Free-living amoebae are protozoa that feed by internalizing energy-rich particles, mainly bacteria [1]. However, some bacteria have adapted to become resistant to amoebal phagocytosis [2]. Some of them have been proposed to be amoebal endosymbionts, including *Amoebophilus asiaticus *[3] and *Candidatus *"Protochlamydia amoebophila" (*Candidatus *"P. amoebophila") [4]. The pathophysiological basis of this specific association is as yet unknown. *Candidatus*. "P. amoebophila" is the only obligate intra-amoebal bacterium whose genomic sequence has been released to date [5].

Recently, we described *Legionella drancourtii *(*L. drancourtii*) within an *Acanthamoeba *sp. amoeba. First named *Legionella*-like amoebal pathogen 12, it was initially thought to be strictly intra-amoebal [6]. To investigate the genetic features associated with its association with amoebae, we sequenced the genome of *L. drancourtii*. In order to identify the genes associated with amoebal parasitism, we herein compared the genome sequences of *L. drancourtii *and *Candidatus *"P. amoebophila" to detect the genes common to both species but absent from other prokaryotes.

## Methods

### Genome sequencing

A two-fold genome sequencing of *L. drancourtii *was performed using the GS20 sequencer (454 Life Sciences, Branford, CT). Open Reading Frame (ORF) prediction was performed using the combination of the GeneMark and GeneMark.hmm programs for prokaryotes [7].

### Orthologous gene determination

The alignment between *L. drancourtii *and *Candidatus *"P. amoebophila" amino acid sequences was performed using the Blastp software. Alignments with a similarity greater than 40% and an ORF coverage greater than 80% were considered significant.

### Specific gene determination

*L. drancourtii *sequences of orthologous ORFs were compared to GenBank using the Blastp software (National Center for Biotechnology Information). ORFs presenting a match with *Candidatus *"P. amoebophila" among the 10 best matches were selected and sorted to remove species redundancy. For selected ORFs, nucleotide sequence was compared to the GenBank nucleotide collection using the Blastn software.

### Phylogenetic analysis

For studied ORFs, amino acid sequences of all matches were recovered from NCBI. Sequence alignment was performed using the Muscle software [8]. Phylogenetic relationships among species were inferred using the Phyml (PHYlogenetic inferences using Maximum Likelihood) software [9].

### Total RNA isolation

*L. drancourtii *[6] was adapted to axenic growth on BCYE medium (BioMerieux, Marcy l'Etoile, France) in a 5% CO_2 _atmosphere at 32°C. RNA was extracted from exponentially-growing bacteria (OD_600 _0.8) using the FastRNA^® ^Pro Blue Kit following the manufacturer's instructions (MP Biomedicals, Illkirch, France). Extracted RNA was resuspended in 100 μl of sterile DNase- and RNase-free water and treated with DNase treatment (Promega, Charbonnieres, France) during 30 min at 37°C.

### Quality and purity control of RNA

RNA quality was controlled using the 2100 bioanalyzer (Agilent, Massy, France). Absence of DNA in the RNA sample was controlled by PCR amplification of the 7-dehydrocholesterol reductase-encoding gene using specific primers (forward primer: 5'-TGACCGTGCTGGTTTTTACA-3', reverse primer: 5'-AAGACGGTAACGGGCTTTTT-3').

### Sterol delta-7 reductase-encoding gene-specific RT-PCR

Transcription of the sterol delta-7 reductase gene was estimated using the SuperScript™ One-Step RT-PCR kit following the manufacturer's instructions (Invitrogen, Cergy Pontoise, France).

### RT-PCR product sequencing

RT-PCR products were purified using NucleoFAST plates (Machery-Nagel, Hoerdt, France) and resuspended in 50 μl of sterile water. Purified products were sequenced using the BigDye^® ^Terminator v3.1 Ready Reaction Mix (Applied Biosystems) as recommended by the manufacturer in a 3130*xl *Genetic Analyzer (Applied Biosystems). RT-PCR product sequences were compared to the 7-dehydrocholesterol reductase-encoding gene of *L. drancourtii *by alignment using the Muscle software [8].

## Results

### *L. drancourtii *sequencing

A preliminary batch of 466,182 sequence reads from *L. drancourtii *was obtained by pyrosequencing. ORF analysis of the 947 contigs greater than 1,000-bp long used for the analysis identified 4,386 resulting ORFs, ranging in length from 15 to 1,999 amino acid residues.

### *In silico *analysis

Among the 4,386 *L. drancourtii *ORFs, 262 putative proteins showed significant alignment with *C*. "P. amoebophila", including two for which this species was the best match. These included a hypothetical Zn-dependent hydrolase of the beta-lactamase fold (data not shown) and a 7-dehydrocholesterol reductase (also referred to as sterol delta-7 reductase [GenBank:FJ197317]) (Table 1). We focused on the sterol reductase because the ten best matches were distributed into two groups: i) a prokaryotic group composed of the two amoeba-associated bacteria *L. drancourtii *and *Candidatus *"P. amoebophila" and the obligate intracellular agent of Q fever, *Coxiella burnetii *(*C. burnetii*), which is able to survive in *Acanthamoeba castellanii *[10], and ii) a eukaryotic group composed of viridiplantae, fungi and metazoa (Table 1).

**Table 1:** Best matches of *L. drancourtii *sterol delta-7 reductase

Organism	Gene Annotation	CovO	CovP	Id
*Candidatus *Protochlamydia amoebophila	Putative 7-dehydrocholesterol reductase	98.61	97.93	58.92
*Coxiella burnetii*	Ergosterol biosynthesis ERG4/ERG24 family protein	99.54	94.51	57.44
*Oryza sativa* (japonica cultivar-group)	Putative sterol delta-7 reductase	98.15	94.22	53.54
*Gossypium hirsutum*	Sterol delta-7 reductase (DWF5)	96.99	95.88	54.18
*Tropaeolum majus*	Sterol delta 7 reductase	98.15	97.47	54.01
*Arabidopsis thaliana*	DWF5	96.99	97.22	52.03
*Oryza sativa* (indica cultivar-group)	7-dehydrocholesterol reductase	59.72	98.85	59.69
*Morus alba*	Sterol delta-7 reductase	66.67	100	56.25
*Nematostella vectensis*	Predicted protein	88.43	95.98	38.74
*Ajellomyces capsulatus*	Conserved hypothetical protein	96.99	100	34.13

Best alignment results from the nr database of *L. drancourtii *sterol delta-7 reductase protein showing the organism of each matching protein, percent of *L. drancourtii *ORF coverage (CovO), percent of matching protein coverage (CovP) and percent identity (Id) of the alignment.

The study of all amino acid sequences matching the *L. drancourtii *protein (73 sequences) showed that this protein exhibited best matches with 7-dehydrocholesterol reductases (also named sterol delta-7 reductases) with similarity rates ranging from 33 to 53%, and then with C14 sterol reductases (similarity rates from 23 to 32%) and C24 sterol reductases (23 to 27%). At the nucleotide level, the sterol reductase-like genes from *L. drancourtii*, *Candidatus *"P. amoebophila"and *C. burnetii*, exhibited a 66% similarity rate.

**Figure 1:**
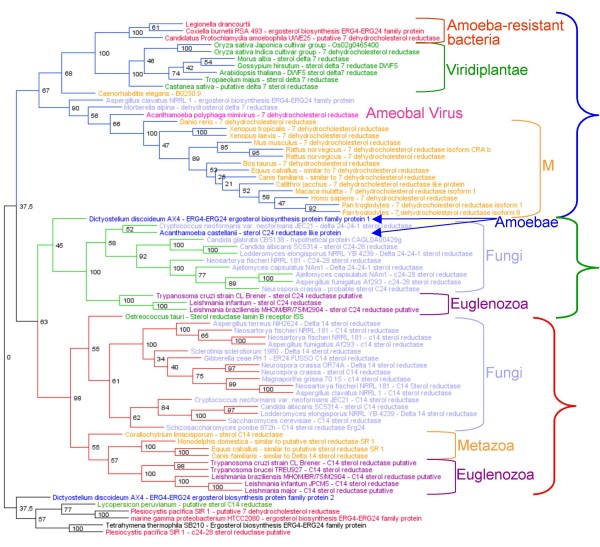
**Phylogenetic analysis of the *L. drancourtii *sterol delta-7 reductase matches**. Unrooted phylogenetic tree of protein sequences matching the *L. drancourtii *sterol delta-7 reductase protein. Three distinct clusters were identified: a cluster contained delta-7 sterol reductases implicated in cholesterol biosynthesis in eukaryotes (blue), a cluster contained C24 sterol reductases implicated in ergosterol biosynthesis in fungi (green) and a third cluster contained C14 sterol reductases implicated in both biosyntheses (red). Bootstrap values are indicated at the nodes.

The phylogenetic analysis showed that the *L. drancourtii *protein is grouped with the 7-dehydrocholesterol reductases cluster (Figure 1), suggesting that this protein is a 7-dehydrocholesterol reductase, and more particularly with *Candidatus *"P. amoebophila" and *C. burnetii*, forming a distinct group of amoeba-resistant bacteria. The strongly supported group (100% support) formed by these three amoeba-resistant bacteria and viridiplantae suggests a vegetal origin of this bacterial 7-dehydrocholesterol reductase.

Two other bacteria have sterol reductases: an unclassified gamma proteobacterium, marine gamma proteobacterium HTCC2080 [11] and a marine bacterium of the *Myxococcales *order, *Plesiocystis pacifica *[12]. However, these proteins are not phylogenetically grouped in the previously identified clusters, suggesting that the 3 amoeba-resistant bacteria are the only bacteria to have a 7-dehydrocholesterol reductase. A genomic search confirmed that this enzyme is not known in other prokaryotes.

### *RT*-PCR analysis

**Figure 2:**
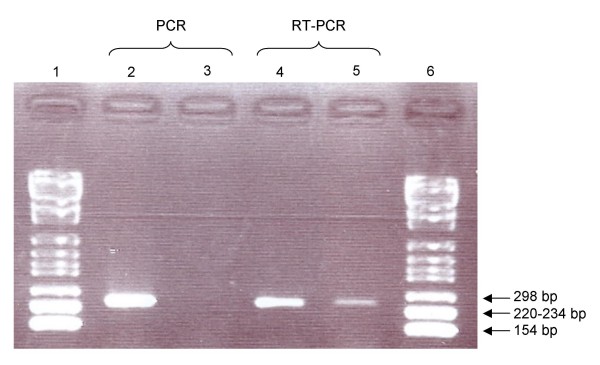
**PCR amplification and RT-PCR of the sterol delta-7 reductase-encoding gene**. Lanes 1 and 6: DNA molecular weight marker VI (Roche); lanes 2 and 4: DNA; lanes 3 and 5: RNA

**Figure 3:**
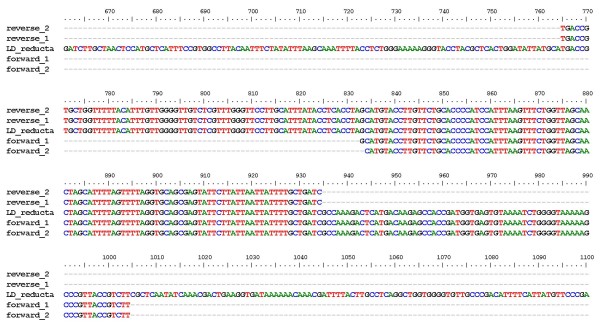
**Sequence alignment of RT-PCR product and sterol delta-7 reductase-encoding gene**. RT-PCR products were sequenced and aligned with the sterol delta-7 reductase-encoding gene of *L. drancourtii *using the Muscle software. "Reverse_1" and "reverse_2" were obtained using the reverse primer, "forward_1" and "forward_2" using the forward primer, "LD_reducta" is the reference sequence obtained from the *L. drancourtii *genome.

RT-PCR from *L. drancourtii *RNA produced a sequence of the expected size (Figure 2). The sequence obtained from the RT-PCR product was identical to that of the 7-dehydrocholesterol reductase-encoding gene of *L. drancourtii *(Figure 3), confirming that it was transcribed. A control PCR performed on the RNA sample proved that there was no DNA contamination.

## Discussion

By comparing the genomes of the intra-amoebal bacteria *Candidatus *"P. amoebophila" and *L. drancourtii *with those of closely related species, we identified a sterol delta-7 reductase, likely acquired from viridiplantae by horizontal gene transfer (HGT). The *L. drancourtii *genome, with a size of 4.2 Mb, appears to be larger than that of sequenced *Legionella pneumophila *strains, with an average size of 3.5 Mb [13,14]. In contrast with other intracellular bacteria in which the intracellular lifestyle is associated with genome reduction [15,16], amoebal endosymbionts appear to have larger genomes than their relatives. The largest difference is in *Candidatus *"P. amoebophila", whose 2.4 Mb genome is about twice as large as the genomes of pathogenic *Chlamydia *species [5]. Another example can be found in *Rickettsia bellii*, able to survive in amoebae, whose genome is the largest among *Rickettsia *species and contains an abundance of amoebal parasite genes, thus showing possible HGT within amoebae [17]. Among viruses, *Acanthamoeba polyphaga mimivirus*, an amoeba virus, also possesses the largest genome (1.18 Mb) of all known viruses [18]. In contrast with obligate intra-cellular bacteria from other eukaryotic cells, which are isolated in their hosts, and thus have a limited ability to exchange genetic material, we hypothesize that amoebae, which feed on bacteria, constitute a favourable place for genetic exchange between intra-amoebal prokaryotes, which may thus have larger genomes that those of other intra-cellular bacteria.

Among orthologous proteins of *Candidatus *"P. amoebophila" and *L. drancourtii*, we discovered a protein similar to enzymes of the sterol reductase family and more particularly similar to sterol delta-7 reductase, otherwise found in most eukaryotes but in only few bacteria. A genomic search confirmed that *L. drancourtii *is the only *Legionella *species to have this protein. Phylogenetically, the *L. drancourtii *protein clustered with the sterol delta-7 reductases of *Candidatus *"P. amoebophila" and *C. burnetii*, also amoeba-resistant, and this group was closely related to sterol delta-7 reductases of viridiplantae supported by a high bootstrap value. The most parsimonious explanation for the presence of this gene in these three amoeba-resistant bacteria is that it was transferred from eukaryotes, more precisely from viridiplantae. An alternative but less likely explanation would be that the gene was lost by all other bacteria.

**Figure 4:**
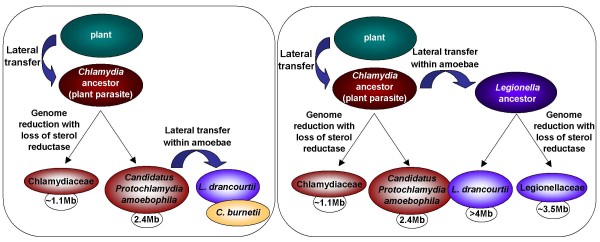
Two hypothetical scenarios to explain the sterol delta-7 reductase gene acquisition by *L. drancourtii*

Inter-kingdom gene transfers have already been reported, notably from bacteria to humans [19] and from bacteria to insects [20]. However, HGT from eukaryotic cells to bacteria is a rare event of particular interest because of the possible influence of such acquired genes on bacterial fitness [21]. In addition, it was shown that some bacterial genomes contain genes from viridiplantae, which may be the case in this study. The most clear-cut example is the eukaryotic ATP/ADP translocase, an enzyme of intracellular symbiont mitochondria and chloroplasts found in *Chlamydiales *and *Rickettsiales*, [22,23]. On the basis of a phylogenetic analysis clustering these proteins with plant homologs, two plausible hypotheses were inferred: i) the ATP/ADP translocase was acquired from a plant by the *Chlamydia *ancestor that might have been a plant parasite and was transferred to rickettsiae [21] or ii) a nuleotide transport protein was invented by the *Chlamydia *ancestor to support its intracellular lifestyle, duplicated, evolved into an ATP/ADP translocase gene and transferred to the *Rickettsia *ancestor and to plants [22,23]. Evidence for gene exchanges between intracellular bacteria of amoebae was shown in the *Rickettsia belli *genome which possesses amoeba-associated bacteria genes [17]. Thus, this transfer from chlamydiae to rickettsiae may have occured within amoebae. We believe that the first hypothesis may apply to the sterol delta-7 reductase-encoding gene, leading to two scenarios (Figure 4): i) the *Chlamydia *ancestor acquired this gene from plants, after a genomic reduction, it was conserved only by *Candidatus *"P. amoebophila" and subsequently transferred to other bacteria sharing the same intra-amoebal biotope or ii) the gene was directly transferred from the *Chlamydia *ancestor to the *Legionella *ancestor within amoebae and after genome reductions, it was conserved only by *Candidatus *"P. amoebophila" and *L. drancourtii*. However, another hypothesis may place the HGT between an as yet unidentified aquatic plant living in the natural habitat of amoebae and intra-amoebal bacteria.

**Figure 5:**
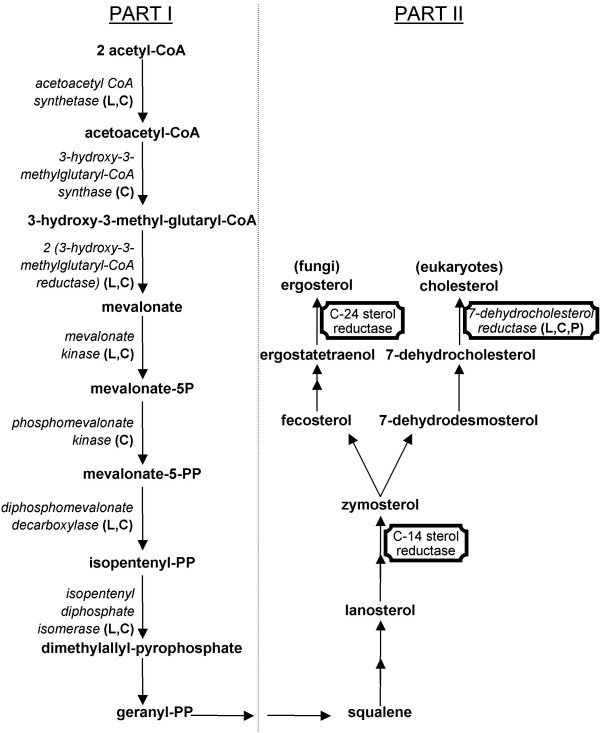
**Eukaryotic sterol biosynthesis**. Ergosterol and cholesterol are synthesized in fungi and all eukaryotes respectively. Enzymes present in *L. drancourtii *(L), *C. burnetii *(C) and *Candidatus *"P. amoebophila" (P) are represented in italics and enzymes present in the previous phylogenetic tree are framed.

In eukaryotes, the sterol delta-7 reductase enzyme catalyses the final step of cholesterol biosynthesis reducing a double bond at carbon 7 of 7-dehydrocholesterol to produce cholesterol (Figure 5), lipid playing important roles in the physiology of eukaryotic organisms [24,25]. This enzyme is a member of the sterol reductase family including the C14 sterol reductase and the C24 sterol reductase, which exhibit high sequence similarities [26] and whose the phylogenetic relationships study showed an organization into three distinct clusters (Figure 1).

Although the role of the sterol delta-7 reductase is not clearly identified in prokaryotes, there is evidence that some intracellular bacteria can interfere with the host cholesterol metabolism. *C. burnetii *upregulates host genes involved in both cholesterol uptake and biosynthesis, inducing cholesterol accumulation necessary for its replication [27]. Moreover, the *C. burnetii *genome has genes predicted to encode enzymes involved in the first part of the sterol biosynthesis (Figure 5) [28]. Comparison of the presence of these genes in the three genomes showed that *Candidatus *"P. amoebophila" has only the sterol delta-7 reductase-encoding gene, while *L. drancourtii *has almost all genes. Some bacteria, like some *Myxococcales *and *Methylococcales*, contain only a portion of enzymatic pathway involved in sterol biosynthesis and as a consequence can synthesize sterols [29,30], however, only these three pathogens have the last enzyme.

We demonstrated that the sterol delta-7 reductase-encoding gene was transcribed in *L. drancourtii*. This result, together with the involvement of this gene in the cholesterol metabolism of eukaryotes, and its presence in *C. burnetii *which is also amoeba-resistant and is cholesterol-dependent, raise the hypotheses that the sterol delta-7 reductase might be functional and play a role in host cholesterol parasitism in *L. drancourtii*.

## Conclusion

In conclusion, by comparing the genomes of two intra-amoebal bacteria with those of their close relatives, we identified a sterol reductase-encoding gene likely acquired from viridiplantae. The role of this gene in bacteria is as yet unknown but it could be involved in host cholesterol parasitism and appear to be linked to intra-amoebal host fitness.

## Competing interests

The authors declare that they have no competing interests.

## Authors' contributions

CM performed the analysis and drafted the manuscript. DR conceived the study and drafted the manuscript. PEF supervised the study and drafted the manuscript. All authors reviewed and approved the final manuscript.
